# Completeness of notifications of accidents involving venomous animals in the Information System for Notifiable Diseases: a descriptive study, Brazil, 2007-2019

**DOI:** 10.1590/S2237-96222023000100002

**Published:** 2023-03-13

**Authors:** Mariana Brito, Ana Caroline Caldas de Almeida, Franciana Cavalcante, Yukari Figueroa Mise

**Affiliations:** 1 Universidade Federal da Bahia, Instituto de Saúde Coletiva, Salvador, BA, Brazil

**Keywords:** Data Accuracy, Health Information Systems, Snake Bites, Scorpion Stings, Spider Bites, Epidemiology, Descriptive

## Abstract

**Objective:**

to describe the completeness of notifications of accidents involving venomous animals held on the Notifiable Health Conditions Information System (SINAN), in Brazil and its macro-regions, from 2007 to 2019.

**Methods:**

we analyzed essential and non-mandatory fields for snakebite, spider bite and scorpion sting notifications, considering the following completeness categories: Excellent (≤5.0% incompleteness), Good (5.0% to 10.0%), Regular (10.0% to 20.0%), Poor (20.0% to ≤50.0%) and Very Poor (>50.0%). Proportional change in completeness between 2007 and 2019 was estimated.

**Results:**

1,871,462 notifications were investigated. The “localized manifestations”, “systemic manifestations”, “case classification”, “case progression” and “zone of occurrence” fields had excellent or good completeness. Completeness was regular or poor for the “schooling” and “race/color” fields. The “occupation” field was predominantly poorly or very poorly filled in. There was a proportional worsening in completeness (PC<0) in most regions for the “zone of occurrence”, “case progression” and “schooling” fields.

**Conclusion:**

completeness of most fields improved, although socioeconomic and occupational fields require more attention.

**Table:** 

Study contributions	
**Main results**	In the period, most of the fields showed improved completeness. Completeness was poorer for fields related to the socio-economic dimension, such as those related to health care provision.
**Implications for services**	Surveillance data support policymaking, and systematic quality monitoring is essential. Recognizing errors and missing information in the production of data informs training strategies for better notification form completion.
**Perspectives**	We recommend studies that explore other dimensions of data quality to reinforce the importance of data reported on the SINAN regarding venomous animals, in order to contribute more robustly to health surveillance, planning and interventions.

## INTRODUCTION

Accidents involving venomous animals are the main cause of human poisoning in Brazil. Their clinical complications can lead to death.^1^ ^,^ ^2^ In 2019, 287,132 accidents involving venomous animals were reported on the Notifiable Health Conditions Information System (*Sistema de Informação de Agravos de Notificação* - SINAN), whereby scorpion sting cases were the most frequent (168,915 records / 58.8%), followed by spider bite cases (13.5%) and snakebite cases (11.3%). The remaining 16.4% of venomous animal notifications were distributed between accidents involving caterpillars, bees, other unspecified poisonous animals, unknown data, or missing information.^3^

The SINAN system stands out among Brazil’s public information systems as a source for studying these accidents, since it has a specific individual investigation form for recording this health condition, given that notification of poisoning is compulsory. This ensures a greater amount of data of epidemiological interest.^4^

The objective of the SINAN system is to collect, transmit and disseminate epidemiological surveillance data. It is fed by the mandatory notification of records of diseases and health conditions contained on the national list of compulsorily notifiable diseases (2017 Ministerial Ordinance).^5^ The system is an important source of surveillance data on venomous animal poisonings in Brazil. The information held on the SINAN is used for epidemiological analysis and strategic research for the Brazilian National Health System (*Sistema Único de Saúde* - SUS), serving to guide public policies and interventions.^6^ Given the importance of these records, it is necessary to constantly evaluate the quality of the data reported on the SINAN. Among the quality dimensions of a surveillance system, completeness of data on reported cases is dependent on the frequency of completion of information on the notification forms.^7^ The objective of this study was to describe the completeness of the data on accidents involving venomous animals and reported on the SINAN in Brazil, between 2007 and 2019.

## METHODS

*Design*

This was a retrospective descriptive study of the completeness of notifications held on the SINAN system for snakebite, spider bite and scorpion sting in Brazil.

*Background*

We analyzed records of cases reported between 2007 and 2019, in municipalities of the 27 Brazilian Federative Units, grouped together according to the five national macro-regions: North, Northeast, Southeast, South and Midwest. The analysis period began in 2007, considering the implementation of the new individual investigation form for accidents involving venomous animals, and ended in 2019, this being the time period necessary for data consolidation.

*Participants*

Cases of individuals injured by venomous animals reported on the SINAN in the defined period were included in the study.

*Variables*

For the purpose of analysis, ten fields were selected as either “essential” or “non-mandatory” with regard to their completion (Box 1), grouped together according to the following factors:

- Fields related to the accident: zone of occurrence (urban; rural; periurban; unknown - essential completion field); time elapsed between bite/sting and receiving health care (in hours: 0-1; 1-3; 3-6; 6-12; 12-24; 24 or more; unknown - essential completion field).
- Fields related to health care: case classification (mild; moderate; severe, unknown - non-mandatory investigation form field); localized manifestations (yes; no; unknown - essential completion field); systemic manifestations (yes; no; unknown - essential completion field); and case progression (cure; death due to accident involving venomous animals; death from other causes; unknown - essential completion field).
- Fields containing social information: race/skin color (White; Black; Asian; mixed race; Indigenous; unknown - non-mandatory investigation form field); schooling (illiterate; incomplete 1^st^ to 4^th^ grade of elementary education; complete 2^nd^ to 4^th^ grade of elementary education; incomplete 3^rd^ to 5^th^ grade of elementary education; complete elementary education; incomplete high school education; complete high school education; incomplete higher education; complete higher education; unknown; not applicable - non-mandatory investigation form field).
- Fields related to work: work-related accident (yes; no; unknown - non-mandatory investigation form field); and occupation (occupation code as per the Brazilian Occupation Classification, household survey version - non-mandatory investigation form field).

*Data source and measurement*

We examined percentage completion of the “essential” and “non-mandatory” fields of the SINAN investigation form for venomous animal accidents, as per item X29 of the International Statistical Classification of Diseases and Related Health Problems (ICD-10).^8^ The “required” fields were not investigated, as requirement prevents the notification from being input to the system with incomplete data, thus systematically resulting in better completion of these fields.^8^

We estimated absolute and relative frequencies of snakebite, spider bite and scorpion sting notifications for each of Brazil’s five macro-regions. Completeness of the fields of the form was investigated according to the completion of valid data, excluding the “unknown” category and values/terms indicating missing data (Table 1), according to the type of accident and reporting region, in the study period.

**Box 1:** - Description of the investigation form fields for venomous animal accidents, on the Notifiable Health Conditions Information System, classified as essential or non-mandatory, 2007-2019

Investigation form field	Type	Description
Fields related to the accident		
Zone of occurrence	Essential	Zone where accident occurred
Time between sting and care	Essential	Time elapsed between accident and arrival at a health service
Work-related accident	Non-mandatory	Accident occurred in workplace or on the way to or from work
Fields related to health care		
Case classification	Non-mandatory	Clinical classification of the injured person at the start of health care
Localized manifestations	Essential	Occurrence of localized clinical manifestations
Systemic manifestations	Essential	Occurrence of systemic clinical manifestations
Case progression	Essential	Clinical outcome of the injured person
Socioeconomic fields		
Race/skin color	Non-mandatory	Ethnic-racial characteristics according to the IBGE^a^ race/skin color classification system
Schooling	Non-mandatory	Grade/year and level of education the person is attending or finished their schooling
Occupation	Non-mandatory	Occupation code as per the Brazilian Classification of Occupations, household survey version

a) IBGE: *Instituto Brasileiro de Geografia e Estatística* (Brazilian Institute of Geography and Statistics).

*Statistical methods*

Data completeness, at the national and regional levels, was analyzed based on a score adapted from Romero & Cunha,^7^ which categorizes completeness as excellent (≤ 5.0% incompleteness), good (5.0% to 10.0% incompleteness), regular (10.0% to 20.0% incompleteness), poor (20.0% to ≤ 50.0% incompleteness), or very poor (> 50.0% incompleteness).

In addition, we determined the proportional change (PC) of completeness by type of accident, year and reporting region. PC was calculated by subtracting percentage completeness in the last year (PerComp-2019) from percentage completeness in the first year (PerComp-2007). The result of this subtraction was divided by the percentage completeness for the first year (PerComp-2007) and multiplied by 100:^9^

PC= ([PerComp-2019 - PerComp-2007] / PerComp-2007) x 100

*Ethical aspects*

The study project was approved by the Research Ethics Committee of the Instituto de Saúde Coletiva of the Universidade Federal da Bahia (CEP-ISC/UFBA): Opinion No. 1.370.415/2015, issued on December 16, 2015.

## RESULTS

A total of 1,871,462 venomous animal accidents were reported on the SINAN between 2007 and 2019. Of these cases, 1,111,300 (59.4%) related to scorpion sting, 386,938 (20.7%) to snakebite, and 373,224 (19.9%) to spider bite. Snakebite stood out in the Northern region, which concentrated 31.6% of cases (n = 122,129), followed by the Northeast region with 26.0% (n = 100,645), the Southeast region with 23.2% (n = 90,004), the Midwest region with 10.3% (n = 39,782), and the Southern region with 8.9% (n = 34,378). The highest percentage of spider bite cases was found in the Southern region (n = 233,785; 62.6%), followed by the Southeast (n = 102,111; 27.4%), Northeast (n = 18,449; 4.9%), North (n = 10,426; 2.8%), and Midwest (n = 8,453; 2.3%). Regarding scorpion stings, the Northeast stood out with 512,533 cases (46.1%), followed by the Southeast (n = 471,564; 42.5%), Midwest (n = 54,843; 4.9%), North (n = 45,415; 4.1%), and South (n = 26,945; 2.4%).

 The North, Northeast and Midwest regions had the highest number of fields with regular, poor or very poor completeness (Table 1). In contrast, the Southern region only had regular or poor completeness for two fields, namely “schooling” and “occupation”, for all accident types. In the Southeast, the regular/poor/very poor degree of completeness varied between two fields, namely “schooling” and “occupation”, regarding scorpion sting cases, as well as varying between five fields - “work-related accident”, “case progression”, “race/skin color”, “schooling” and “occupation” - with regard to snakebite cases.

With the exception of the field corresponding to “case progression”, all other fields related to care (“localized manifestations”, “systemic manifestations” and “case classification”) had higher degrees of completeness (excellent and good) in all regions. Still with regard to the “case progression” field, regular completeness was found for snakebite accidents among the regions, with the exception of the Southern region, where completeness for this field was good. Completeness of the “case progression” field was regular in the Midwest region for all three types of accident, and also in the Northeast region with regard to spider bite (Table 1).

**Table 1:** - Distribution of the completeness percentage and score^a^ for the snakebite, spider bite and scorpion sting investigation form fields, on the Notifiable Health Conditions Information System (N = 1,871,462), Brazil, 2007-2019

Health condition	Investigation form field	Completeness, %											
		Brazil		North		Northeast		Southeast		South		Midwest	
		%	Score	%	Score	%	Score	%	Score	%	Score	%	Score
Snakebite	Zone of occurrence	96.5	excellent	97.0	excellent	96.6	excellent	95.4	excellent	96.6	excellent	97.0	excellent
	Time between sting and care	93.2	good	94.0	good	90.7	good	93.5	good	95.8	excellent	94.2	good
	Work-related accident	84.5	regular	82.7	regular	80.3	regular	87.9	regular	92.4	good	86.4	regular
	Case classification	93.8	good	94.5	good	91.0	good	94.7	good	96.4	excellent	94.0	good
	Localized manifestations	97.7	excellent	98.0	excellent	96.3	excellent	98.4	excellent	98.5	excellent	97.8	excellent
	Systemic manifestations	93.6	good	93.9	good	91.3	good	94.7	good	96.2	excellent	93.6	good
	Case progression	87.1	regular	86.3	regular	84.3	regular	89.6	regular	91.4	good	87.6	regular
	Race/skin color	90.6	good	95.2	excellent	85.3	regular	88.5	regular	96.2	excellent	90.2	good
	Schooling	65.1	poor	71.4	poor	58.6	poor	59.4	poor	75.9	poor	66.3	poor
	Occupation	49.5	very poor	50.0	poor	46.3	very poor	49.1	very poor	60.7	poor	47.0	very poor
Spider bite	Zone of occurrence	97.2	excellent	97.4	excellent	96.4	excellent	96.1	excellent	97.7	excellent	97.3	excellent
	Time between sting and care	92.7	good	92.7	good	83.7	regular	91.4	good	94.0	good	89.6	regular
	Work-related accident	92.0	good	86.3	regular	78.1	poor	88.2	regular	95.3	excellent	84.4	regular
	Case classification	96.8	excellent	94.5	good	92.1	good	95.1	excellent	98.1	excellent	94.2	good
	Localized manifestations	98.7	excellent	98.0	excellent	96.1	excellent	98.3	excellent	99.1	excellent	97.5	excellent
	Systemic manifestations	96.3	excellent	94.1	good	91.2	good	95.1	excellent	97.4	excellent	93.0	good
	Case progression	92.6	good	90.0	good	86.8	regular	91.4	good	93.8	good	89.3	regular
	Race/skin color	91.3	good	95.2	excellent	77.5	poor	86.8	regular	94.5	good	82.8	regular
	Schooling	72.1	poor	70.7	poor	53.1	poor	60.4	poor	79.2	poor	62.1	poor
	Occupation	53.2	poor	50.2	poor	38.8	very poor	46.0	very poor	58.1	poor	41.3	very poor
Scorpion sting	Zone of occurrence	97.1	excellent	97.7	excellent	96.5	excellent	97.6	excellent	97.8	excellent	97.2	excellent
	Time between sting and care	90.3	good	93.5	good	86.0	regular	94.4	good	96.3	excellent	90.5	good
	Work-related accident	87.3	regular	85.4	regular	82.5	regular	92.7	good	96.0	excellent	82.1	regular
	Case classification	96.0	excellent	94.9	good	94.8	good	97.4	excellent	98.1	excellent	95.4	excellent
	Localized manifestations	97.6	excellent	97.9	excellent	96.3	excellent	98.9	excellent	99.1	excellent	97.1	excellent
	Systemic manifestations	93.8	good	94.1	good	91.1	good	96.6	excellent	97.6	excellent	92.6	good
	Case progression	93.4	good	91.6	good	92.0	good	95.6	excellent	96.6	excellent	89.1	regular
	Race/skin color	82.4	regular	94.8	good	74.4	poor	90.0	good	94.4	good	76.3	poor
	Schooling	59.0	poor	72.7	poor	51.2	poor	65.2	poor	82.1	regular	54.9	poor
	Occupation	44.2	very poor	50.8	poor	38.4	very poor	49.0	very poor	68.1	poor	39.5	very poor

a) Completeness score adapted from Romero & Cunha: excellent (≥ 95%); good (< 95% to ≥ 90%); regular (< 90% to ≥ 80%); poor (< 80% to ≥ 50%); very poor (< 50%).

As for the fields related to social factors, “schooling” showed poor completeness in all five regions of the country for snakebite and spider bite; on the other hand, the Southern region had regular completeness for scorpion sting. Among the social factors, the “race/skin color” field was the only one that had good or excellent completeness for all three accident types, in the Northern and Southern regions; however, in the Northeast region, the “race/skin color” field had regular or poor completeness for the three accidents investigated (Table 1).

As for the factors relating to the accident, completeness of the “time elapsed between sting and treatment” field (time elapsed between the accident and the medical care provided) was poor for spider bite notifications in the Northeast and Midwest regions, as well as for scorpion sting notifications in the Northeast region. In contrast, the “zone of occurrence” field had excellent completeness in all regions, for all accidents (Table 1).

Regarding the work-related fields, the completeness of the “occupation” field varied between poor and very poor, in all regions of Brazil and for all types of accidents studied. The complementary “work-related accident” variable had regular completeness, except for Southern region notifications, for which completeness of this field was classified as good (snakebite) and excellent (spider bite and scorpion sting), Southeast region notifications, for which it had good completeness (scorpion sting), and Northeast region notifications, for which spider bite notification completeness was poor (Table 1).

Trends of improvement or worsening in the degree of field completeness were also seen by calculating proportional change (PC). The Southern region, which had the highest number of fields with good or excellent completeness, also had the highest number of negative proportional changes (PC < 0.0) in field completion, throughout the period studied, for all types of accidents. On the contrary, in the Southeast region the degree of completeness only worsened for one field, “case progression”, for snakebite and spider bite notifications (Table 2).

**Table 2:** - Distribution of proportional change in completeness for the snakebite, spider bite and scorpion sting investigation form fields, on the Notifiable Health Conditions Information System (N = 1,871,462), Brazil, 2007-2019

Health condition	Investigation form field	Completeness (change: %)																	
		Brazil			North			Northeast			Southeast			South			Midwest		
		2007	2019	Change	2007	2019	Change	2007	2019	Change	2007	2019	Change	2007	2019	Change	2007	2019	Change
Snakebite	Zone of occurrence	96.9	96.5	-0.4	97.9	97.0	-1.0	97.4	96.6	-0.9	95.0	95.7	0.7	97.5	96.2	-1.4	96.7	96.8	0.1
	Time between sting and care	92.6	93.9	1.4	93.1	95.3	2.4	91.2	91.6	0.4	92.7	93.8	1.2	95.4	95.9	0.5	91.6	95.5	4.3
	Work-related accident	81.0	85.9	6.0	78.8	86.2	9.4	78.2	80.8	3.4	82.2	89.4	8.7	90.6	90.9	0.3	81.2	88.6	9.1
	Case classification	92.6	94.6	2.2	91.1	96.8	6.2	90.3	91.5	1.3	94.1	95.5	1.5	97.2	94.1	-3.2	93.9	95.1	1.3
	Localized manifestations	96.7	98.4	1.8	93.1	98.7	6.0	96.2	97.6	1.5	97.1	99.0	2.0	97.9	98.6	0.8	95.3	98.5	3.4
	Systemic manifestations	89.6	95.4	6.5	88.6	96.4	8.8	87.6	93.2	6.3	91.5	96.2	5.2	94.5	96.1	1.7	86.7	96.1	10.9
	Case progression	88.8	85.3	-4.0	85.3	87.0	2.0	89.0	81.3	-8.7	90.1	87.6	-2.8	92.8	87.8	-5.4	91.0	84.2	-7.5
	Race/skin color	90.3	94.2	4.4	95.2	97.1	2.0	87.8	90.9	3.6	85.5	93.1	9.0	95.5	97.0	1.6	87.6	94.4	7.7
	Schooling	69.7	65.9	-5.5	75.3	71.9	-4.6	67.6	58.2	-13.9	60.2	63.6	5.8	80.3	73.5	-8.4	69.9	68.2	-2.5
	Occupation	43.9	61.3	39.6	45.4	64.3	41.7	41.2	58.2	41.3	41.5	62.4	50.5	59.2	63.5	7.2	33.8	56.2	66.4
Spider bite	Zone of occurrence	97.5	97.1	-0.4	98.1	97.6	-0.5	96.8	96.6	-0.2	95.8	96.8	1.0	98.0	97.3	-0.7	95.3	98.0	2.8
	Time between sting and care	90.8	93.1	2.5	93.3	92.5	-0.8	81.4	85.2	4.7	88.9	92.6	4.2	91.7	94.5	3.1	85.6	90.5	5.8
	Work-related accident	90.4	92.3	2.1	86.8	89.1	2.7	73.7	80.3	8.9	84.8	91.5	7.9	92.6	94.8	2.3	85.9	88.6	3.1
	Case classification	97.1	96.8	-0.3	94.0	95.2	1.3	91.7	91.9	0.2	95.6	96.6	1.1	97.7	97.7	-0.1	94.9	95.0	0.1
	Localized manifestations	98.6	98.7	0.1	97.4	98.3	1.0	96.9	97.0	0.1	97.3	98.8	1.6	99.1	99.0	-0.1	98.1	98.3	0.2
	Systemic manifestations	95.5	96.8	1.4	92.8	95.7	3.2	89.1	93.7	5.1	92.1	97.1	5.4	96.7	97.1	0.4	89.8	96.1	7.0
	Case progression	88.8	92.3	3.9	91.3	89.6	-1.9	90.8	85.0	-6.4	93.9	91.4	-2.7	87.3	94.0	7.7	92.2	89.7	-2.7
	Race/skin color	88.6	93.6	5.7	91.6	96.6	5.5	76.2	86.0	13.0	79.4	91.7	15.5	91.5	95.8	4.8	79.7	89.2	12.0
	Schooling	72.1	73.1	1.5	76.4	70.8	-7.3	54.6	56.4	3.3	55.4	67.1	21.2	76.9	79.3	3.1	68.4	65.9	-3.6
	Occupation	48.5	58.5	20.6	41.9	64.2	53.1	31.0	47.9	54.7	37.6	56.6	50.5	52.3	61.3	17.2	32.4	46.7	44.1
Scorpion sting	Zone of occurrence	97.6	97.4	-0.1	97.9	98.1	0.3	98.1	97.0	-1.1	96.9	97.8	0.9	98.3	97.6	-0.7	96.2	97.5	1.4
	Time between sting and care	87.3	92.1	5.6	94.2	93.6	-0.7	82.7	89.1	7.8	91.7	95.1	3.6	97.4	96.3	-1.1	90.5	90.9	0.5
	Work-related accident	82.4	90.4	9.8	80.8	88.8	9.9	78.3	86.9	11.1	87.5	94.5	8.1	95.5	95.6	0.0	82.1	85.8	4.55
	Case classification	96.1	96.6	0.5	91.3	96.6	5.8	96.2	95.1	-1.2	96.5	98.0	1.6	98.0	98.1	0.2	95.2	96.2	1.1
	Localized manifestations	97.2	98.4	1.2	97.3	98.6	1.3	96.5	97.4	0.9	98.0	99.4	1.4	99.1	99.3	0.2	96.6	98.2	1.7
	Systemic manifestations	92.4	95.3	3.1	90.4	96.7	7.0	91.7	92.7	1.1	93.7	97.9	4.4	97.1	97.9	0.9	89.0	94.5	6.2
	Case progression	95.5	93.4	-2.3	89.8	91.7	2.1	96.0	91.5	-4.7	95.9	95.9	0.0	96.9	95.8	-1.1	93.1	89.1	-4.3
	Race/skin color	79.2	89.5	13.0	94.6	96.5	2.0	74.4	84.2	13.2	83.3	95.2	14.3	92.2	96.7	4.9	74.6	80.9	8.4
	Schooling	57.1	63.5	11.4	80.7	74.7	-7.4	48.0	55.4	15.5	63.6	71.0	11.5	87.6	81.0	-7.6	65.6	56.2	-14.3
	Occupation	34.5	52.3	51.4	44.8	64.3	43.6	30.6	43.7	42.7	37.1	60.4	62.7	57.6	68.3	18.6	32.6	44.0	35.3

As for the factors related to care, the “systemic manifestations” field showed no loss in completeness for all regions and for all types of accident. Completeness of the “localized manifestations” field only became poorer (0.11%) with regard to spider bite in the Southern region. In turn, “case classification” field completeness became poorer in the South for snakebite and spider bite, and in the Northeast for scorpion sting. Similarly, the “case progression” field predominantly expressed worsening proportional change, with improvements only in the North region for snakebite, in the Southern region for spider bite, and in the Northern and Southeast regions for scorpion sting. 

Among the fields related to social characteristics, “race/skin color” showed improved completion for all types of accidents and in all regions; on the contrary, “schooling” worsened with regard to completion of snakebite cases - except in the Southeast, where it improved by 5.8%. With regard to spider bite, completeness of the “schooling” field worsened in the North and Midwest regions, while completeness of the “schooling” field with regard to scorpion sting field worsened in these regions and as well as in the Southern region. 

As for the factors related to the accident, we found that completion of the “zone of occurrence” field worsened in relation to all accidents in the Northeast and Southern regions; and in the Northern region for snakebite and spider bite accidents. Completeness also worsened in relation to the “time elapsed between bite and treatment” field for spider bite cases in the North, and for scorpion sting in the North and South. Completeness of the work-related fields (“occupation” and “work-related accident”) improved for all accidents in all five regions (Table 2).

**Figure 1:**
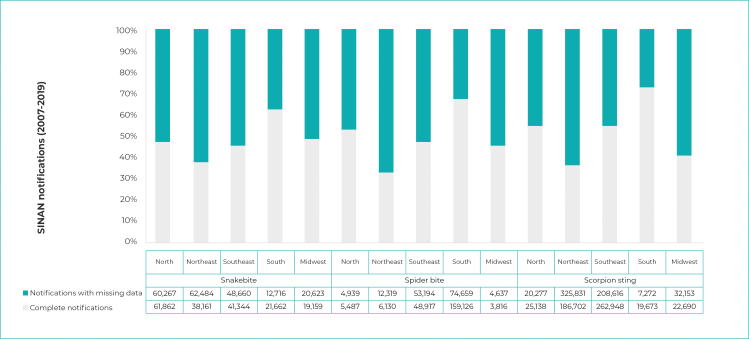
- Distribution of the completeness of snakebite, spider bite and scorpion sting cases notified on the Notifiable Health Conditions Information System (N =1,871,462) by Brazilian macro-region, 2007-2019

## DISCUSSION

The notification completeness classification pattern, based on the score adapted from Romero and Cunha,^7^ varied little for snakebite, spider bite and scorpion sting cases. In the period studied, an improvement in the completeness of almost all the fields was found, except for “schooling”, “case progression”, and “zone of occurrence”.

The analysis of venomous animal accident notifications recorded on the SINAN from 2007 to 2019, showed variations in the degree of completeness of the fields evaluated for snakebite, spider bite and scorpion sting cases, whereby greater completeness was found for the fields related to the accident itself and care, although the proportional changes in the completeness of the fields related to the accident showed a worsening trend in some regions. We highlight the poor and very poor completeness of the fields related to socioeconomic characteristics, especially those related to work (occupation) and schooling, with low completeness in all Brazilian regions. 

As for the limitations of this study, it is worth noting that only one dimension of data quality was addressed, namely completeness. Thus, it is recommended that research be conducted to evaluate other dimensions of record quality, including health condition notification consistency, reliability, validity and coverage. Additionally, the consolidation of the data held on the SINAN was limited to the inclusion of the most recent data on the accidents. Although using information relating to the initial and final year of the period in order to estimate proportional change enables the change in the time interval to be shown, it does not enable analysis of the completeness of the data for each year in the period.

There was variation in the completeness of the venomous animal accident notifications, considering the two parameters adopted. The score proposed by Romero & Cunha^7^ allowed us to highlight, in general, the fields and dimensions for which lack of completion implied greater loss of information, especially the socioeconomic fields, including “occupation” and “schooling”. However, despite being widely used in studies that evaluate the completeness of national information systems, this score does not allow us to estimate variations in the degree of incompleteness of some fields^10^ because the percentage completeness of a field may vary, either upwards or downwards, even within the limits of the initial completeness category. This finding can be seen in the “zone of occurrence” field, which showed worsening of completeness that was only perceptible after estimating percentage change (PC). Calculating PC, used as a complementary measure, proved to be effective for estimating the quality of the recording of fatal accidents at work in Brazil between 2007 and 2012.^9^

Although studies of the relationship between work and the health-disease process are relatively longstanding, knowledge of the influence or impact of these factors has improved and gained visibility; however, problems such as underreporting and poor data quality still pose challenges.^11^

In this study, the completeness of the fields related to work was poorer than that of all the other fields evaluated. Despite the improvement in completeness found between the first and last years of the period analyzed, the corresponding percentage change was not sufficient to enable achievement of better completion scores, the classification of which ranged from regular to very poor.

In Brazil, health information systems have improved in recent decades through the implementation of strategies such as the publication of ministerial ordinances,^12^ the integration of information between systems, and the use of linkage techniques, among other actions. However, these improvements are still insufficient.^13^

Problems with the quality of occupational data records are not exclusive to the SINAN. A study intended to evaluate the quality of information regarding the variables held on the Live Birth Information System (*Sistema de Informações sobre Nascidos Vivos* - SINASC) in relation to the year 2002, identified problems with the “maternal occupation” field with regard to definition, coding, completeness and consistency.^14^ Another study, which gathered data for the period 2000-2014 from the SINASC system and from the Mortality Information System (*Sistema de Informações sobre Mortalidade* - SIM) in the state of Rio Grande do Sul, found flaws in data input to this system, which the authors attributed to lack of clarity in the manuals, plurality of people responsible for filling out the declarations, and greater attention paid to some variables to the detriment of others.^15^

On March 20, 2020, the Ministry of Health published Ordinance No. 458/2020, which provides for the inclusion and mandatory completion of fields on health information systems related to type of occupation and economic activity.^12^ With the advent of this norm, an increase is expected in the completeness of the “occupation” and “work-related accident” fields of venomous animal accident investigation forms on the SINAN.

The occupational dimension is an important social determinant of health regarding the occurrence of accidents involving venomous animals, such as snakebite cases, classically related to agricultural occupations, which bring workers closer to snakes in the rural environment.^16^ Like snakebites, *Phoneutria* and *Latrodectus* spider bites and scorpion stings can also be related to agricultural activity, because they are animals that can lodge in food and agricultural supplies stores, where they find small prey (for example, insects, other arachnids) and a protective space.^1^ ^,^ ^17^ Scorpion sting cases and *Loxosceles* spider bite cases are also commonly related to domestic activities and building works, especially during handling of undergrowth, accumulated debris and organic waste.^1^ ^,^ ^18^

Unlike the work-related fields, classified as “non-mandatory”, the “essential completion fields referring to “localized and systemic manifestations”, had good and excellent completeness, and an increase in the proportion of completion, in the period from 2007 to 2019, for all the diseases studied and for all geographic regions. The Ministry of Health classifies as “mandatory”, “essential” or “non-mandatory” the completion of the fields of the venomous animal poisoning investigation form on the SINAN.^8^ This classification could result in the mistaken understanding that more attention should be paid to the completion of some fields rather than others, while in fact, they correspond to fields which if not completed prevent the notification form from being concluded on the system.^19^

Despite “case progression” being classified as an “essential” field, its classification was regular and its completeness worsened in some regions of the country, in the period studied, for all three diseases investigated. On the other hand, the “case classification” field, the completion of which is considered “non-mandatory”, achieved excellent and good scores, and improved completeness in most regions. It is worth noting that information lost due to blank fields may also reflect the degree of importance (of a subjective nature) given by the professional responsible for notification.^20^ Case classification directly determines the number of vials of antivenom serum to be administered, this being information necessary for the clinical management of the injured person.^21^

Unfortunately, loss of information on the clinical progression of injured individuals can make it difficult to monitor fatal and non-fatal outcomes, implying problems in the availability of health services for treatment.^22^ In the current scenario of antivenom serum rationing, information on the clinical progression of injured people is essential for monitoring the stock of these immunobiologicals and the measures taken in this scenario, such as changes in the algorithms for serum therapy, and stockout of bothrops antivenom in Brazil in 2021.^23^

Treatment of accidents is closely dependent on the etiological agent involved. Each poison has different mechanisms of action and, therefore, demands specific treatment.^23^ ^,^ ^1^ The Ministry of Health defines guidelines for the identification of venomous animals involved in accidents, based on the symptoms and clinical signs presented during injured people’s stay in health services.^21^ Furthermore, the time elapsed between the accident and admission to the care unit is extremely important information for the correct administration of treatment, since this time interval is directly related to the severity and clinical progression of the injured person.^24^ Information lost when filling out these fields conceals the real scenario of health care for victims, especially in a situation of antivenom shortage,^23^ this being a potentially serious problem, especially among children and the elderly, who are more prone to complications arising from venomous animal bite poisoning.^21^

Among the national information systems, the SINAN has the largest number of variables of interest for investigating venomous animal bite poisoning.^4^ Despite its limitations and divergences with other systems, such as the SIM system, assessing the quality of SINAN data on venomous animal accidents is essential. Adequate completion of these data allows reliable information to be generated on the health-disease process, to be used in the monitoring and definition of intervention priorities by health service managers.^25^

Data quality is essential for the production of reliable information. The way in which data is recorded affects the entire chain of information processes, from registration to storage and analysis.^26^ ^,^ ^27^ In Brazil, the quality of the records held on the different health information systems is not uniform and, therefore, it is necessary to consider differences in the quality of filling out the forms, since the number of victims varies substantially according to the region of the country and the type of venomous animal involved. 

Health surveillance actions use compulsory notification data to assist in the analysis of the health situation and in decision making. Inequalities in data loss can conceal information about these accidents and consequently underestimate vulnerabilities.^28^ A study evaluating the quality of health information system data in Brazil found inter- and intra-regional, and interstate inequalities in the evaluation of data quality. Furthermore, there is a concentration of studies dedicated to specific places in the Southeast and Northeast regions.^28^

These differential information losses are especially complex in venomous animal accident records, since the distribution of the biodiversity of medically important species in the country is not homogeneous and regional particularities may be obscured by these missed opportunities for information.^1^ Furthermore, data losses, particularly those related to socioeconomic and occupational characteristics, make it difficult to identify populations vulnerable to accidents. Although classically, agricultural workers have poorer schooling levels and recognize themselves as being of Black race/skin color (Black/mixed race), the loss of this information makes it impossible to analyze this characteristic from a social perspective.^29^ This problem is evident in data produced by most epidemiological studies of these accidents, which are limited to describing individual attributes without considering social determinants.^30^ Inconsistencies in information are also relevant because they affect its reliability, by generating false health diagnoses, as well as compromising the evaluation and definition of intervention measures for these events.^25^

Health information system data are currently the only source of knowledge on the magnitude of accidents caused by venomous animals in Brazil, a country of continental dimensions and which is a hotspot for the diversity of venomous animal species of medical importance on a global level. It is necessary to raise awareness among health professionals about the importance of recording socioeconomic, occupational and accident-related characteristics on the SINAN, so as to enable robust analysis of data on the situation, for planning, intervention and evaluation of health actions according to SUS guidelines, in the approach to and care of accidents caused by venomous animals and in the treatment of affected individuals.
